# The differential effects of CBD and CBDA on viability and mRNA expression in colorectal cancer cells

**DOI:** 10.1186/s42238-026-00391-2

**Published:** 2026-01-16

**Authors:** Christine Heinzle, Kathrin Geiger, Reinhard Ertl, Eva Maria Brandtner, Andreas Leiherer, Stella Gaenger, David Schmidmayr, Heinz Drexel, Axel Muendlein

**Affiliations:** 1 Vorarlberg Institute for Vascular Investigation and Treatment (VIVIT), Feldkirch, Austria; 2 Medical Central Laboratories, Feldkirch, Austria; 3 VetCore Facility for Research, University of Veterinary Medicine, Vienna, Austria; 4 Private University in the Principality of Liechtenstein, Triesen, Liechtenstein; 5 Better Plants R&D GmbH, Bludenz, Austria; 6 Vorarlberger Landeskrankenhausbetriebsgesellschaft, Feldkirch, Austria; 7 Drexel University College of Medicine, Philadelphia, PA USA; 8 Vorarlberg Institute for Vascular Investigation and Treatment (VIVIT), Molecular Biology Laboratory, Dornbirn, Austria Stadtstrasse 33, 6850

**Keywords:** CBD, CBDA, *Cannabis sativa*, Plant extract, Colorectal cancer, Entourage effect, RNA sequencing

## Abstract

**Background:**

Cannabinoids have attracted significant attention for their potential therapeutic application in cancer research. However, recent studies have reported antitumor activity of cannabidiolic acid (CBDA)—the acidic precursor of CBD—in breast cancer cells, involving modulation of cyclooxygenase signaling. To our knowledge, no investigations have examined the effects of CBDA on RNA expression and signaling pathways in colorectal cancer (CRC) cells. Therefore, we aimed to investigate the effects of CBD, CBDA, and a CBDA-rich *Cannabis sativa* (C.s). extract on the growth and gene expression in CRC cell lines.

**Methods:**

We assessed cell viability and clonogenic growth of the CRC cell lines HCT116 and DLD1 following treatment with pure CBD, pure CBDA, a CBDA-rich C.s. extract (CBDA/CBD ratio 20:1), and a corresponding mixture of pure CBDA/CBD. RNA sequencing was performed to analyze differentially expressed genes (DEGs) and the cell signaling pathways affected by these treatments.

**Results:**

Of all tested compounds, CBD exhibited the strongest cytotoxic effect in both cell lines, whereas CBDA demonstrated minimal toxicity, particularly in HCT116 cells. Furthermore, we observed a greater inhibitory effect of the CBDA-rich C.s. extract on HCT116 cell growth compared to the CBDA/CBD mixture. RNA sequencing analysis revealed that CBD had the most pronounced impact on gene expression, while CBDA had the least. Notably, treatment with the C.s. extract resulted in a higher number of DEGs than the CBDA/CBD mixture in HCT116. Gene expression analysis indicated an upregulation of the Wnt and Hippo signaling pathways following CBD treatment. Additionally, CBDA, CBD/CBDA (1:20), and the C.s. extract primarily induced metabolic processes in DLD1 cells, suggesting a distinct metabolic response.

**Conclusion:**

Our findings showed that CBD exerts stronger effects on cell survival and gene expression in CRC cells than CBDA, which showed only limited activity. Moreover, the CBDA-rich C.s. extract exhibited greater efficacy than the CBDA/CBD mixture. More research is needed to further elucidate the impact of cannabinoids on CRC cell biology and signaling pathways.

**Supplementary Information:**

The online version contains supplementary material available at 10.1186/s42238-026-00391-2.

## Introduction

Colorectal cancer (CRC) is one of the most prevalent malignancies worldwide, being the third of all diagnosed cancers. Despite advancements in screening and preventive colonoscopies as well as in conventional treatments such as surgery, chemotherapy, radiotherapy, and targeted therapy, CRC remains associated with the second-highest mortality rate amongst all cancers (Bray et al., 2024). Therefore, there is an urgent need for novel therapeutic approaches that also include phytochemicals derived from medicinal plants. Among these, cannabinoids from the *Cannabis sativa* (C.s.) plant have emerged as promising candidates. Several studies have analyzed the impact of cannabinoids and C.s. extracts on CRC in vivo and in vitro (Cherkasova et al., 2024; Ladin et al., 2016; Nallathambi et al., 2018).

To date cannabinoid drugs containing synthetic Δ−9- tetrahydrocannabinol (THC) have been approved for the management of chemotherapy-induced side effects, such as nausea and vomiting, whereas cannabidiol (CBD)-containing drugs are approved for treatment of rare forms of epilepsy but, so far, not for indications in cancer treatment (U.S. Food and Drug Administration 2023). CBD, which, unlike THC, does not produce psychotomimetic or euphorigenic effects (Alexander and Joshi 2019; Gülck and Møller 2020; Rock et al., 2021), also exhibits antioxidative, analgesic, and anxiolytic properties and may help reduce depressive symptoms and sleep disorders (O’Brien 2022; Formato et al., 2020). Moreover, in vitro studies suggest that CBD exerts anticancer effects by inducing cell cycle arrest, promoting apoptosis, and inhibiting chemotaxis, cell migration, cell adhesion, angiogenesis, invasion, and metastasis (O’Brien 2022). However, its therapeutic role in cancer pathogenesis in patients remains controversial.

The acidic precursor of CBD, cannabidiolic acid (CBDA), has been studied far less extensively than CBD. One contributing factor is its limited chemical stability: CBDA readily undergoes non-enzymatic decarboxylation to CBD upon exposure to heat, light, or prolonged storage (Wang et al., 2016; Citti et al., 2018). Nevertheless, in vivo studies indicate that CBDA can persist under physiological conditions and reach measurable systemic concentrations (Tittle et al., 2022). When administered as part of a cannabis extract, CBDA plasma levels have been reported to be substantially higher compared with administration as a single isolated molecule, due to interactions with other cannabis constituents such as cannabigerol or Δ9-tetrahydrocannabinol that inhibit intestinal drug efflux transporters (Anderson et al., 2021). In general, it’s presence at a substantially higher concentrations in raw or minimally processed cannabis extracts, suggests that its biological effects may be more relevant in vivo than previously assumed (Kim et al., 2024).

Several factors make CBDA an interesting and relevant target in context of cancer. In preclinical models, CBDA has shown greater efficacy than CBD in attenuating nausea and vomiting, but clinical confirmation in cancer patients is not yet available (Bolognini et al., 2013). In breast cancer cells, CBDA has been reported to inhibit cyclooxygenase (COX) activity by modulating various pathways and regulatory factors (Suzuki et al., 2017; Takeda et al., 2014; Ruhaak et al., 2011; Hirao-Suzuki et al., 2020; Takeda et al., 2017). COX signaling—particularly COX-2–mediated prostaglandin production—plays a pivotal role in colorectal carcinogenesis by promoting tumor cell proliferation, inflammation-driven progression, angiogenesis, and metastatic potential (Jin et al., 2023). These findings highlight the potential of CBDA for the development of novel CBDA-based therapeutic drugs; however, research on CBDA remains limited. Moreover, it remains uncertain whether CBDA exhibits distinct mechanisms of action when used as an isolated compound compared to its presence within a plant extract.

The present study aimed to assess the effectiveness of CBDA in two distinct colon cancer cell lines in comparison with CBD focusing on cell viability and mRNA expression. Using a C.s. extract with a 20:1 ratio of CBDA to CBD, we further compared the effects of a CBDA-rich extract to those of a corresponding pure CBDA/CBD mixture with the same ratio in order to assess potential differences in their biological activity.

## Materials and methods

### Cannabis extract preparation

The C.s. extract was freshly prepared using dried flowers of a CBD-enriched *Cannabis sativa* variety (Lemon Haze CBD), cultivated under a controlled indoor laboratory environment at Better Plants R&D GmbH (Bludenz, Austria). In this context, the dried flowers were mixed with 96% ethanol at a 1:4 weight ratio for 5 min using a blender. During this procedure, the mixing container was placed in a water bath to cool the ingredients. The mixture was then filtered, and stored for 2 months at room temperature, protected from light to minimize CBDA decarboxylation, until it was used for treatment of human CRC cell lines as described below.

The C.s. extract was analyzed by Kalb Analytik GmbH (Feldkirch, Austria) using high-performance liquid chromatography (HPLC) with UV/VIS detection to quantify its main cannabinoids, and gas chromatography (GC) with flame ionization detection (FID) to profile 40 different terpenoids, immediately before it was used for the cell culture experiments. The original extract contained 0.14% CBD and 2.45% CBDA. A table of all measured cannabinoids and terpenoids is available in the additional files (*Additional File 1a and 1b*).

### Cannabinoids

CBD and CBDA were both purchased as pure substances from Merck (Darmstadt, Germany; product codes: PHL85705 and PHL85839, respectively) and dissolved in 96% ethanol. Pure CBD and CBDA were mixed at a 1:20 ratio to match the proportion observed in the C.s. extract, hereafter referred to as CBD/CBDA (1:20). Aliquots of the resulting solutions were stored at −20 °C until use.

### Cell lines

HCT116 and DLD1 human CRC cell lines were kindly provided by Prof. Brigitte Marian (Center of Cancer Research, Medical University of Vienna, Austria) and authenticated by Microsynth AG (Balgach, Switzerland). Both cell lines were maintained under standard conditions at 37 °C in a 5% CO_2_ atmosphere and cultured in DMEM/Ham’s F12 (1:1) medium (Thermo Fisher Scientific, Waltham, MA, USA) supplemented with 10% fetal calf serum (FCS; Thermo Fisher Scientific). Routine testing for mycoplasma contamination was carried out (MycoSPY®, Biontex, Munich, Germany).

### Cell viability assay

To determine cell viability, neutral red (Merck) uptake into the cells was measured. For this, 10,000 cells per well were seeded into a 24-well plate in quadruplicates and cultured for 24 h. The medium was then replaced by a cell culture medium, containing either 0 µM, 5 µM, 10 µM, 15 µM, 20 µM, or 25 µM of CBD, CBDA, CBD/CBDA (1:20), or C.s. extract (concentration based on its CBD/CBDA content). Each treatment medium was equilibrated with an adequate amount of 96% ethanol to ensure consistent solvent concentrations across all treatment groups. After 72 h, the treatment medium was replaced by a neutral red solution (50 µg neutral red/mL medium), and cells were incubated for 2 h. The cells were then washed with phosphate-buffered saline (PBS; Thermo Fisher Scientific), fixed with 70% ethanol + 1% acetic acid for 5 min, and the absorption was measured at 562 nm, with a reference wavelength of 620 nm. Results from at least three independently performed neutral red assays were pooled.

### Colony formation assay

Single cells were seeded at very low density (100 or 200 cells per well) into 6-well plates in cell culture medium containing 10 µM of either CBD, CBDA, CBD/CBDA (1:20), or C.s. extract. Medium containing an equivalent concentration of 96% ethanol served as the control group. After 24 h of incubation, the medium was replaced with fresh DMEM/Ham’s F12 (1:1) containing 10% FCS, and unattached cells were removed. After 7 days, the colonies were washed with PBS and fixed with ice-cold methanol for at least 20 min. The cells were washed again with PBS and then stained with a 0.01% crystal violet solution. Colonies were counted, and colony size was measured using ImageJ software (ImageJ, U.S. National Institutes of Health, Bethesda, Maryland, USA). Results from at least three independently performed colony formation assays were pooled.

### RNA extraction

A total of 1 × 10⁶ cells were seeded into 10 cm Petri dishes. After 24 h, the medium was removed, and the cells were treated with 10 µM of either CBD, CBDA, CBD/CBDA (1:20), C.s. extract, or ethanol for the control group for 48 h. Each experiment was performed three times to obtain biological triplicates.

After incubation, the cells were washed with cold PBS and lysed with 1 mL of Trizol (TRI-Reagent®, Merck). Cell lysates were stored at −80 °C until being sent to the VetCore Facility for Research (University of Veterinary Medicine, Vienna, Austria) for RNA extraction and sequencing. RNA was extracted with the Direct-zol RNA Miniprep Kit (Zymo Research, Irvine, CA, USA) according to the manufacturer’s recommended protocol. RNA concentrations and RNA integrity numbers (RIN) were assessed on a 4200 TapeStation system with the RNA ScreenTape assay (Agilent, Santa Clara, CA, USA). All samples showed RIN values > 8 and were used for sequencing analysis.

### RNA sequencing

RNA sequencing (RNA-seq) was performed by the next generation sequencing facility at Vienna BioCenter Core Facilities, member of the Vienna BioCenter, Austria. Sequencing libraries were prepared from 200 ng RNA using the CORALL mRNA-Seq V2 kit (Lexogen, Vienna, Austria) following the manufacturer’s protocol with 14 PCR cycles. Library quality control was done with the High Sensitivity D1000 ScreenTape assay on the 4200 TapeStation (Agilent). The samples were sequenced on one lane of a NovaSeq X 10B flowcell (Illumina, San Diego, CA, USA) implementing 150-bp single-end reads. Quality filtering of the raw sequence reads was done with Trimmomatic 0.38 (Bolger et al., 2014). Adapter sequences, low-quality bases (Phred score < 20), and reads shorter than 50 nucleotides were removed. The filtered reads were imported into CLC Genomics Workbench 23.0.5 (Qiagen, Hilden, Germany) and mapped to the human reference genome GRCh38/hg38 using the default mapping parameters (mismatch cost = 2, insertion cost = 3, deletion cost = 3, length fraction = 0.8 and similarity fraction = 0.8). For differential expression analysis, data from treated samples were compared pairwise to controls. Genes were considered as differentially expressed when showing false discovery rate (FDR) corrected *p*-values < 0.05 and log_2_ fold change (FC) >|1.0|.

### Functional enrichment analysis

Up- and downregulated genes were analyzed for enriched Gene Ontology (GO) terms using ShinyGO 0.81 (Ge et al., 2019) with the following settings: species “human”, pathway size “min = 10, max = 5000”, and an FDR cut-off of 0.05. REVIGO 1.8.1 [http://revigo.irb.hr/] was used to reduce the redundancy of the enriched GO terms (Supek et al., 2011). Additionally, functional enriched pathway analysis was performed using the web-based tool ‘integrated Differential Expression and Pathway analysis’ (iDEP2.01), [http://bioinformatics.sdstate.edu/idep/]. Gene set enrichment analysis based on GO terms and Kyoto encyclopedia of genes and genomes (KEGG) pathways was performed using a cutoff of FDR < 0.05 and log_2_ FC >|1.0|. For more stringent analysis and to filter out noisy genes with high FDRs, genes with an FDR > 0.05 were removed before pathway analysis. Principal component analysis (PCA) plots were generated with ggfortify (Tang et al., 2016).

### Statistical analysis

Statistical differences in cell viability were analyzed using IBM SPSS Statistics, version 29.0.1.0 (IBM Corp, Armonk, NY, USA), and the half maximal inhibitory concentration (IC50) values were calculated in GraphPad Prism, version 10.2.2 (GraphPad Software, San Diego, CA, USA). Colony formation data are presented as mean ± standard deviation (SD). Normal distribution was assessed using Kolmogorov–Smirnov and Shapiro–Wilk tests. Statistical differences between treated groups were assessed using unpaired Student's t-test for parametric data and Mann–Whitney U test for nonparametric data in GraphPad Prism, version 10.2.2. Volcano plots for RNA sequencing exploratory data analysis were generated in GraphPad Prism, version 10.2.2. Venn diagrams were generated using Venny 2.1.0, applying a cutoff of FDR < 0.05 and log₂FC >|1.0| [http://bioinfogp.cnb.csic.es/tools/venny/index.html].

## Results

### Dose–response of CBD, CBDA, and C.s. extract in colorectal cancer cells

**Fig. 1:**
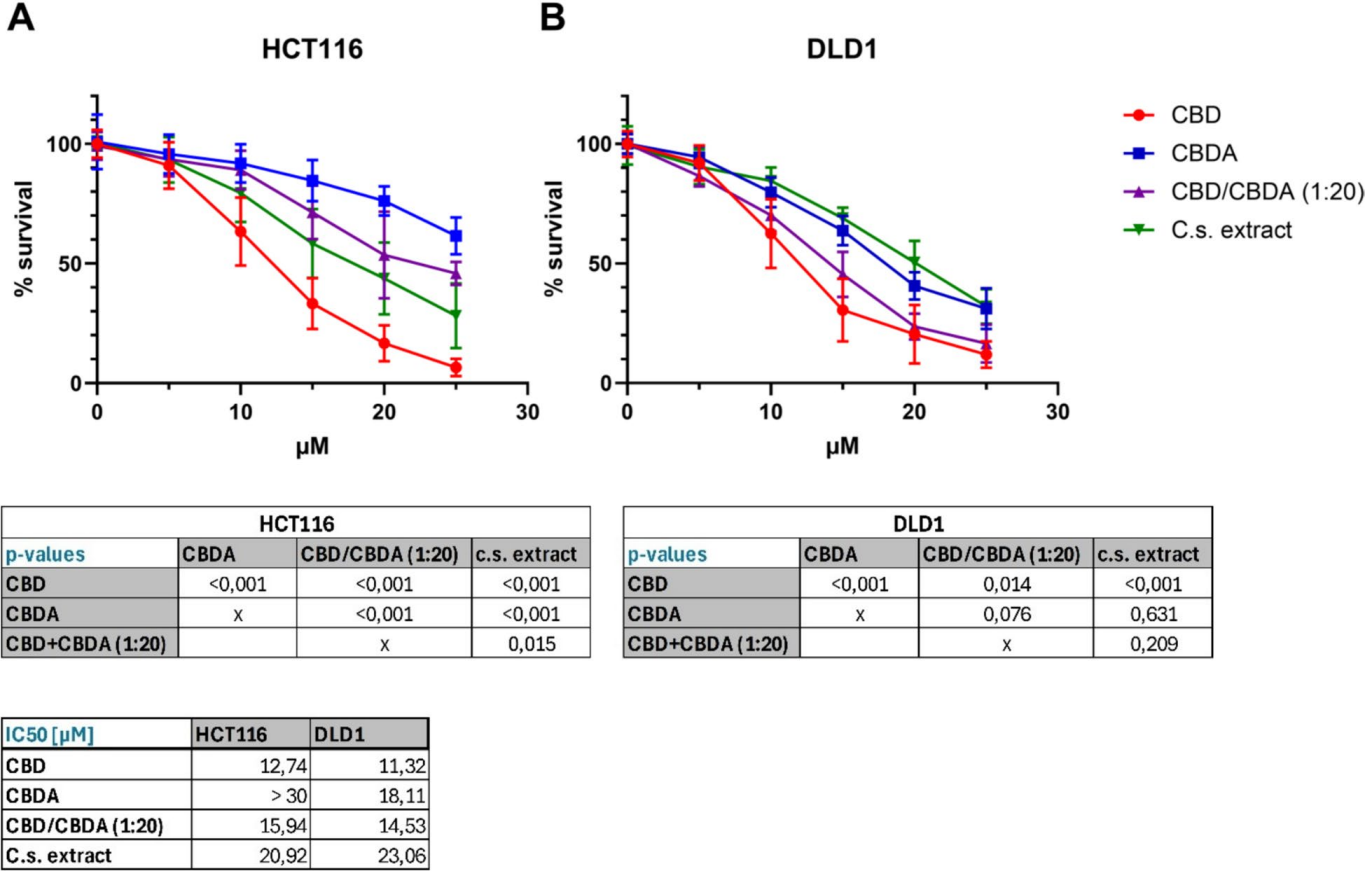
Dose–response curves for cell viability following treatment with CBD, CBDA, CBD/CBDA (1:20), and C.s. extract. HCT116 **A** and DLD1 **B** cells were treated for 72 h with increasing concentrations (1–30 µM) of either CBD, CBDA, CBDA/CBD (20:1), or C.s. extract. Cell viability was assessed using the neutral red uptake assay. Data are presented as % of survival (mean ± SD) from three independent experiments (*n* = 3)

The dose–response for CBD, CBDA, CBD/CBDA (1:20), and C.s. extract was evaluated in the two CRC cell lines, HCT116 and DLD1. The corresponding dose–response curves are shown in Fig. 1. Treatment with CBD resulted in a highly significant decrease in cell viability (*p* = 0.014 compared with DLD1 cells treated with CBD/CBDA (1:20), and *p* < 0.001 compared with all other treatment groups in both cell lines), with an IC50 of 12.74 µM for HCT116 and 11.32 µM for DLD1 cells.

In HCT116 cells (Fig. 1A), dose–response curves for CBDA, CBD/CBDA (1:20), and C.s. extract differed highly significantly from each other with a *p*-value of < 0.001 (except of CBD/CBDA (1:20) compared with C.s. extract with *p* = 0.015), with CBDA being the least effective substance, followed by CBD/CBDA (1:20) and C.s. extract. Surprisingly, CBD/CBDA (1:20) showed a lower IC50 (15.94 µM) compared to C.s. extract treatment (20.92 µM). The IC50 for CBDA could not be determined within the tested concentration range (up to 30 µM) and is therefore reported as > 30 µM. In DLD1 cells (Fig. 1B), there was no statistically significant difference between the dose–response curves of CBDA, CBD/CBDA (1:20), and C.s. extract, but IC50 values demonstrated higher toxicity for CBD/CBDA (1:20) (14.53 µM) compared to CBDA (18.11 µM) and C.s. extract (23.06 µM).

Based on the findings of the dose–response study, we continued using a concentration of 10 µM for all four treatment groups in the subsequent experiments.

### Cannabinoids affect clonogenic growth of colorectal cancer cells

**Fig. 2:**
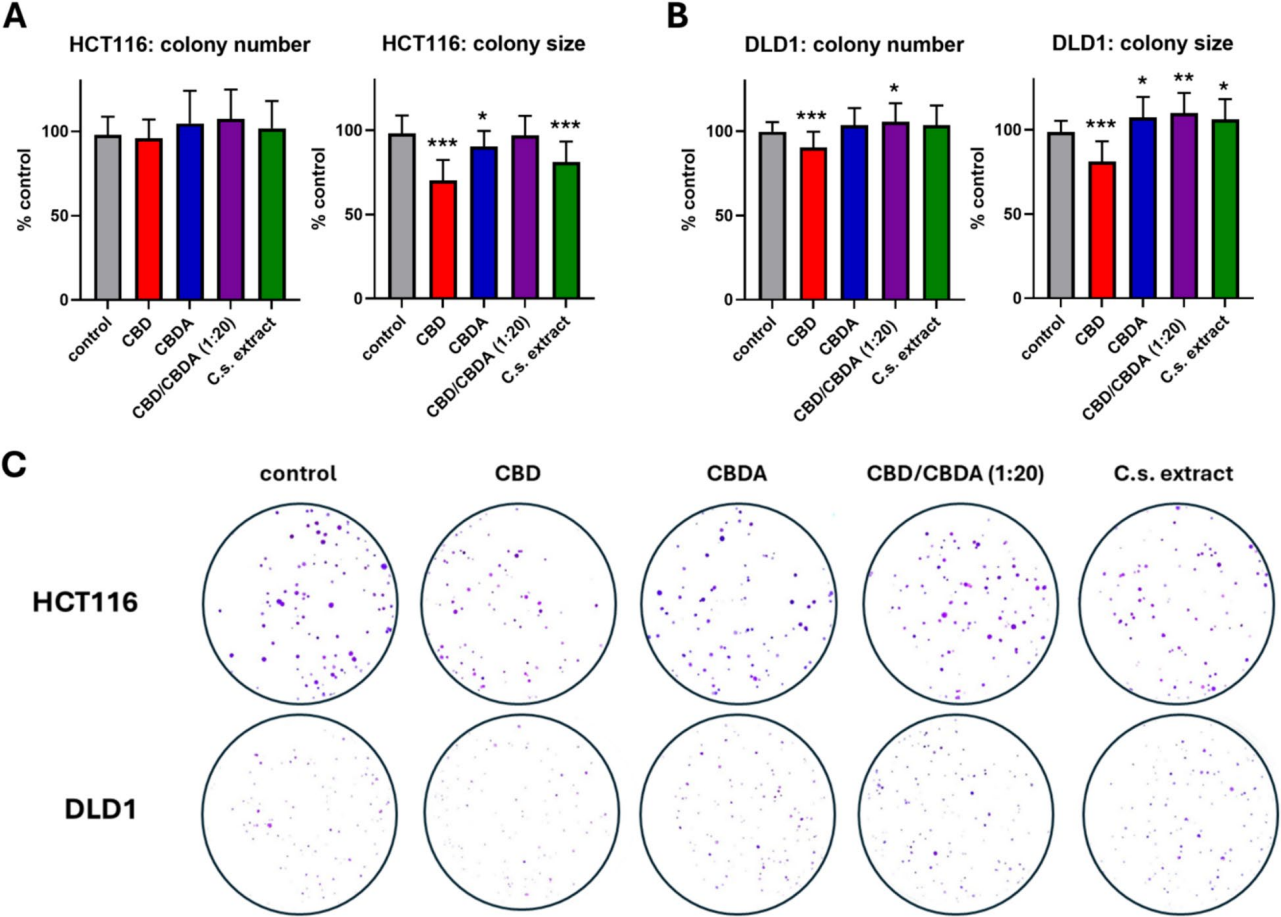
Clonogenic growth of colorectal cancer cells under CBD, CBDA, CBD/CBDA (1:20), or C.s. extract treatment. Quantification of colony number and average colony size in HCT116 **A** and DLD1 **B** cells following treatment with 10 µM of either CBD, CBDA, CBD/CBDA (1:20), or C.s. extract administered at the time of seeding. Cells were treated for 24 h, followed by 7 days of incubation under standard culture conditions to allow colony formation. Results are normalized to control cells treated with solvent only and presented as mean ± SD from three independent experiments (*n* = 3). *, **, and, *** indicate statistically significant differences compared to the control at *p* ≤ 0.05, < 0.01, and < 0.001, respectively. **C** Representative images of colony formation assays from HCT116 and DLD1 cells under the same treatment conditions

Cell adhesion and clonogenic growth of HCT116 and DLD1 cells were assessed following treatment with CBD, CBDA, CBD/CBDA (1:20), or C.s. extract by analyzing colony number and colony size, respectively (Fig. 2A, B, C). In the HCT116 cell line, treatment with cannabinoids showed no effect on single-cell adhesion. In the DLD1 cell line, however, the presence of CBD during cell seeding significantly reduced the number of colonies compared to the ethanol-treated control. Surprisingly, pure CBD/CBDA (1:20) significantly increased the number of colonies (*p* = 0.035). The other treatment groups showed no marked difference from the control group.

Clonogenic outgrowth was assessed by measuring colony size, revealing a highly significant reduction with CBD treatment in both cell lines (*p* < 0.001) compared to the control. In HCT116 cells, treatment with C.s. extract also significantly reduced colony growth (*p* < 0.001), whereas CBDA significantly increased colony size (*p* = 0.016). No difference in colony size was observed between cells treated with CBD/CBDA (1:20) and the control. In DLD1 cells, colony growth was significantly increased by the treatment with CBDA, CBD/CBDA (1:20), and C.s. extract compared to the control group, with *p*-values of 0.018, 0.003, and 0.040, respectively.

### RNA-seq reveals a higher number of differentially expressed genes after treatment with CBD compared to CBDA

RNA-seq analysis was performed to identify genes and pathways that were differentially expressed in CRC cell lines treated with either ethanol (control), CBD, CBDA, CBD/CBDA (1:20), or C.s. extract. RNA-seq generated an average of 41.5 million raw reads per sample, out of which an average of 38.7 million clean reads remained after quality filtering. The mapping rate to the reference genome was > 95% for all samples.

**Fig. 3:**
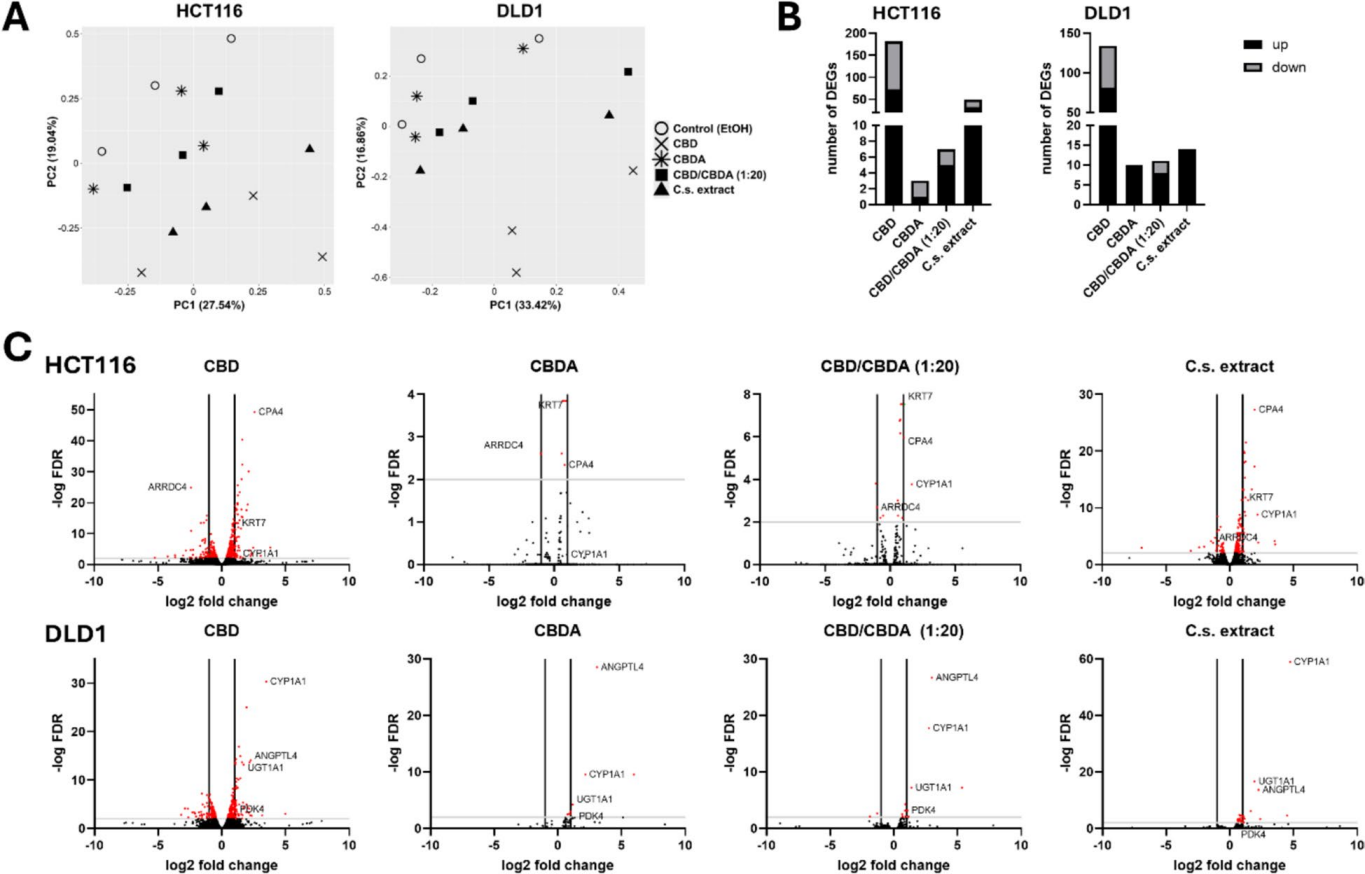
Principal component analysis and differentially expressed genes after treatment with cannabinoids and C.s. extract. **A** Principal component analysis plots of RNA-seq data from HCT116 and DLD1 colorectal cancer cells treated for 48 h with 10 μM of CBD, CBDA, CBD/CBDA (1:20), or C.s. extract. **B** Number of differentially expressed genes (DEGs) identified in each treatment condition, separated into upregulated (black) and downregulated (gray) genes, in HCT116 and DLD1 cells. **C** Volcano plots illustrating DEGs in each treatment group for both cell lines. Red dots above the horizontal threshold line indicate genes with false discovery rate (FDR) < 0.05. Genes outside the two vertical lines represent those with a log_2_ fold change (log_2_FC) >|1.0|

PCA plots in Fig. 3A illustrate gene expression patterns by representing the first and second principal components (PC1 and PC2) capturing the largest sources of variation in the dataset. For HCT116 cells, PC1 and PC2 accounted for 27.5% and 19.0% of the total variation, respectively, while for DLD1 cells, they explained 33.4% and 16.9% of the total variation. In both cell lines, CBD samples were clearly distinct from the ethanol control group, indicating that the greatest changes in gene expression occurred after CBD treatment. In HCT116, the C.s. extract-treated cells were also clearly distinct from the control group, whereas CBDA and CBD/CBDA (1:20) appeared to have a somewhat smaller impact. Treatment clusters were more tightly grouped in the DLD1 cell line for CBDA, CBD/CBDA (1:20), and C.s. extract treatments, suggesting that DLD1 cells might exhibit a less pronounced response to these treatments.

Figure 3B shows an overview of the number of differentially expressed genes (DEGs) in both cell lines after treatment with CBD, CBDA, CBD/CBDA, and C.s. extract, applying a cut-off of FDR ≤ 0.05 and a log_2_FC >|1.0|. CBD treatment resulted in the highest number of DEGs across both cell lines (182 DEGs for HCT116 and 134 DEGs for DLD1). By contrast, the number of DEGs in CBDA-treated cells was very small, with 3 DEGs in HCT116 cells and 10 DEGs in the DLD1 cell line. Also, treatment with CBD/CBDA (1:20) showed very low numbers of DEGs (7 DEGs for HCT116 and 11 DEGs for DLD1). HCT116 cells showed 49 DEGs in the presence of C.s. extract, whereas in DLD1 cells, only 14 genes were differentially expressed. All DEGs for both cell lines are listed in the additional files (*Additional file 2*).

Volcano plots for DEGs of HCT116 and DLD1 after treatment are shown in Fig. 3C, highlighting the most strongly regulated genes*.* Some DEGs could be found in almost all treatment groups like for example, carboxypeptidase 4 (CPA4), arrestin domain-containing 4 (ARRDC4), keratin 7 (KRT7), and cytochrome P450 family 1 subfamily A member 1 (CYP1A1) for HCT116 and CYP1A1, angiopoietin-like 4 (ANGPTL4), UDP glucuronosyltransferase family 1 member A1 (UGT1A1), and pyruvate dehydrogenase lipoamide kinase isozyme 4 (PDK4) for DLD1 cells.

### CYP1A1 and ANGPTL4 are differentially expressed in both CRC cell lines when treated with CBD, CBDA, CBD/CBDA (1:20), or C.s. extract

**Fig. 4:**
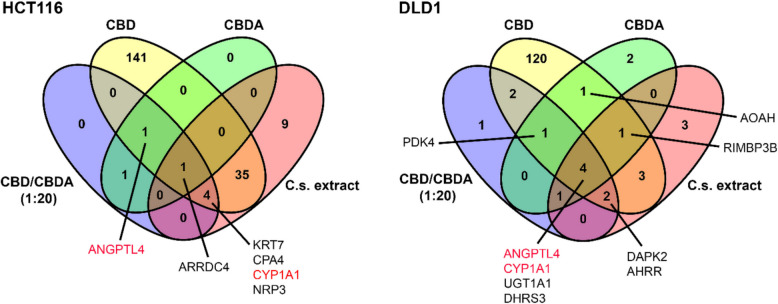
Venn diagram from differentially expressed genes after treatment with cannabinoids or C.s. extract. Venn diagrams display the number of overlapping differentially expressed genes (DEGs) identified in HCT116 and DLD1 colorectal cancer cells after treatment with 10 μM of either CBD, CBDA, CBD/CBDA (1:20), or C.s. extract. DEGs were defined by a false discovery rate (FDR) ≤ 0.05 and an absolute log_2_ fold change (log_2_FC) >|1.0|. Genes shown in red are differentially expressed in both cell lines and across nearly all treatment groups

To compare DEGs between the different treatment groups, Venn diagrams were created (Fig. 4). In HCT116 cells treated with either CBD or CBDA, only two genes were differentially expressed by both groups, namely ARRDC4 and ANGPTL4. Furthermore, ARRDC4 was differentially expressed in all four treatment groups across this cell line. For CBD/CBDA (1:20)- and C.s. extract-treated HCT116 cells, there was an overlap of five DEGs, namely KRT7, CPA4, CYP1A1, ARRDC4, and neuropilin 2 (NRP2).

When analyzing the DLD1 cell line, there was a communality of four DEGs for all treatment groups: ANGPTL4, CYP1A1, UGT1A1, and short-chain dehydrogenase/reductase 3 (DHRS3). Apart from these four DEGs, both CBD- and CBDA-treated DLD1 cells also overexpressed PDK4, acyloxyacyl hydrolase (AOAH), and RIMS Binding Protein 3B (RIMBP3B) statistically significantly compared to the control. When comparing CBD/CBDA (1:20) with C.s. extract treatment in DLD1, death-associated protein kinase 2 (DAPK2), and aryl hydrocarbon receptor repressor (AHRR) genes were also differentially expressed. In both cell lines, ANGPTL4 was found to be differentially expressed when treated with CBD, CBDA, and CBD/CBDA (1:20). Additionally, CYP1A1 was differentially expressed in both cell lines in all treatment groups except the CBDA treatment in HCT116.

### HCT116 cells show higher mRNA expression of CBD binding targets CB2, TRPV1, TRPV2, and TRPM8 than DLD1 cells

**Fig. 5:**
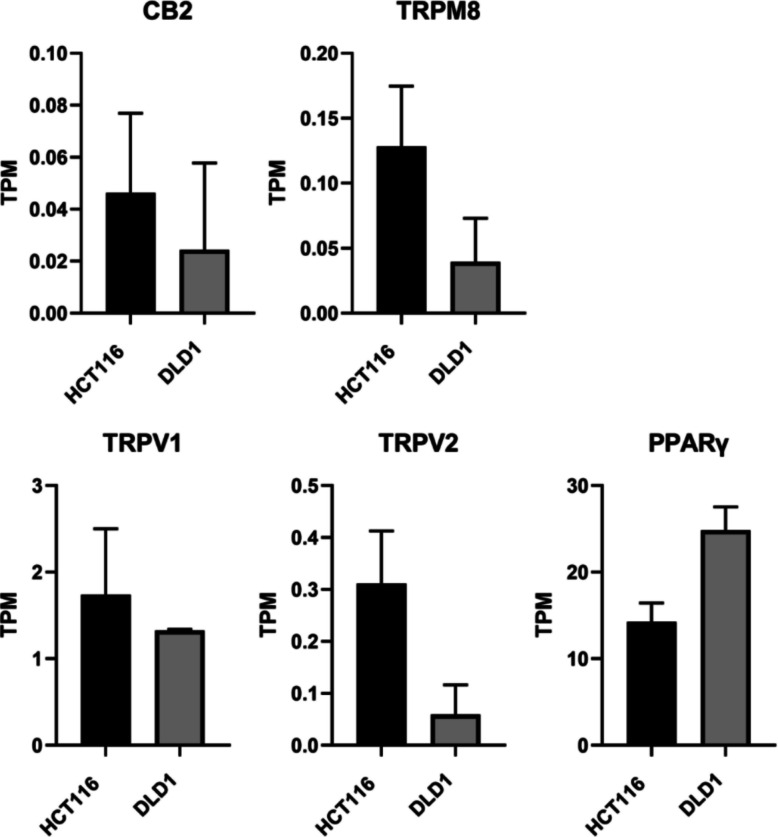
mRNA expression of cannabinoid and cannabinoid-related receptors in colorectal cancer cell lines. mRNA expression levels of CB2, TRPM8, TRPV1, TRPV2, and PPARγ in HCT116 and DLD1 cells were determined by RNA sequencing. Expression values are presented as transcripts per million (TPM). Data represent the mean ± SD from three independent biological replicates (*n* = 3). Statistical comparisons between HCT116 and DLD1 cells were performed for all targets; however, none of the differences reached statistical significance, and therefore no statistical annotations are shown

From our RNA-sequencing data, we analyzed the mRNA expression levels of CB1 and CB2 receptors, as well as other known CBD-binding targets — including transient receptor potential (TRP) cation channels (TRPV1, TRPV2, TRPM8, and TRPA1), G protein-coupled receptor 55 (GPR55), serotonin 1 A receptor (5-HT1A), and peroxisome proliferator-activated receptor gamma (PPARγ) (Seltzer et al., 2020; Etemad et al., 2022), to better understand the sensitivity of HCT116 and DLD1 cells to CBD. As shown in Fig. 5, HCT116 cells displayed higher mRNA expression levels of CB2, TRPV1, TRPV2, and TRPM8 compared to DLD1 cells, whereas DLD1 cells exhibited higher PPARγ expression. However, none of these differences reached statistical significance. Consistent with this, the expression levels of CB2, TRPV2, and TRPM8 were generally very low with TPM values below 1. Notably, mRNA expression of CB1, TRPA1, GPR55, and 5-HT1A was undetectable in both cell lines.

### CBD and C.s. extract induce cell motility and cytoskeletal arrangements in HCT116 and metabolic processes in DLD1 cells

GO enrichment analysis of the RNA-seq data set showed substantial differences in biological functions and biological processes among the treatment groups. In HCT116, treatment with 10 µM CBD resulted in a significant upregulation of 62 biological processes and downregulation of 72 biological processes. After C.s. extract treatment, 18 processes were upregulated, while no downregulation was observed. CBDA and CBD/CBDA (1:20) treatment groups showed no stimulation of any biological processes under the predefined FDR cut-off (*Additional file 3*). In the DLD1 cell line, all four treatment groups resulted in significant upregulation of biological processes (33 GO terms for CBD, 21 GO terms for CBDA, 29 GO terms for CBD/CBDA (1:20), and 17 GO-terms for C.s. extract). CBD treatment also resulted in a significant downregulation of 16 different biological functions, whereas no downregulation of any biological process was observed for the other substances (*Additional file 4*).

**Fig. 6:**
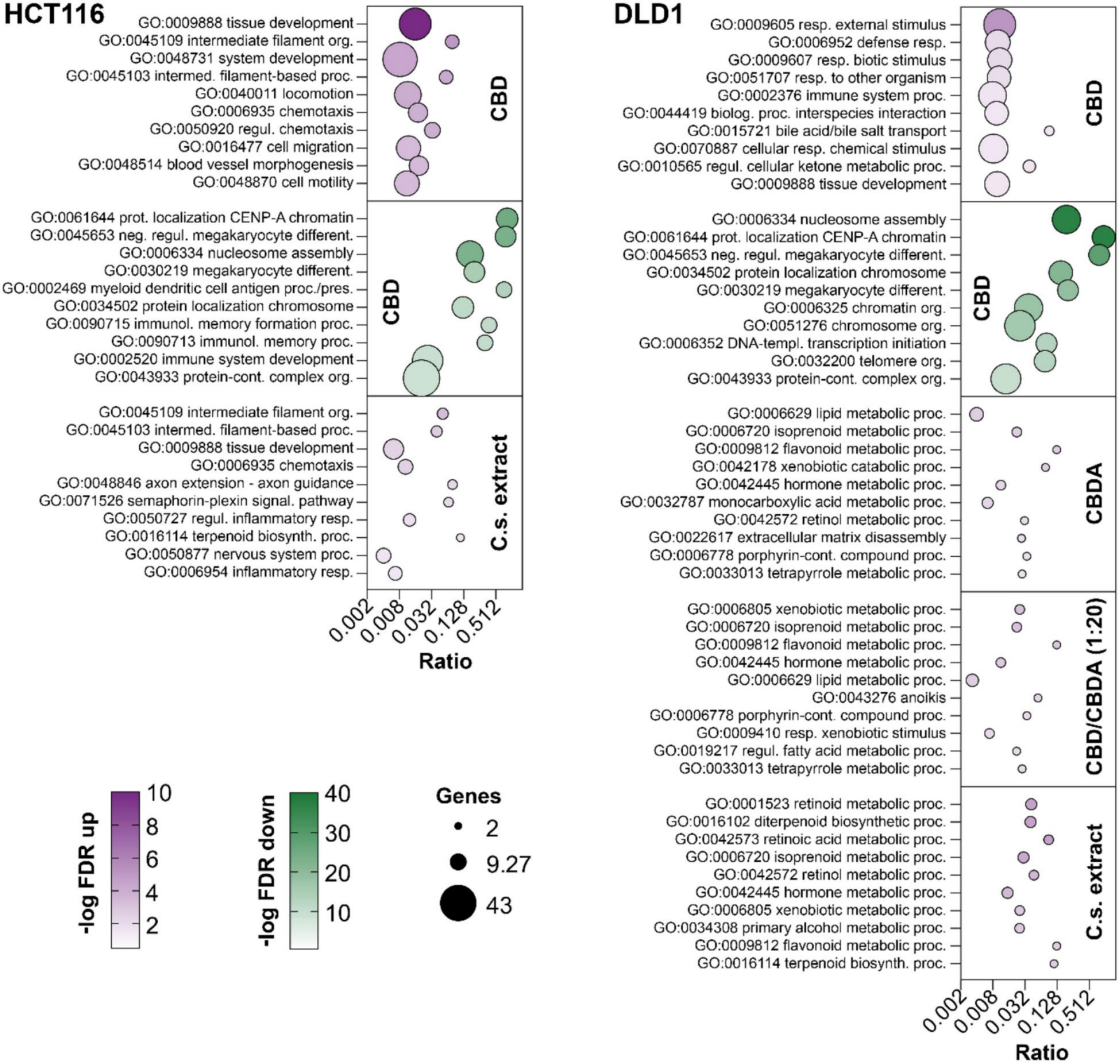
Bubble plot of the top 10 upregulated and downregulated Gene Ontology terms for biological processes. This figure presents the top 10 significantly upregulated (magenta) and downregulated (green) Gene Ontology (GO) terms for biological processes in HCT116 and DLD1 cells after treatment with 10 μM of either CBD, CBDA, CBD/CBDA (1:20), or C.s. extract. Each bubble represents a GO term, with the position on the x-axis indicating the ratio of regulated genes to the total number of genes associated with that term. The color intensity reflects the significance level based on the FDR, where darker colors denote more significant changes. Bubble size corresponds to the number of genes involved in each GO term. Data are grouped by cell line (HCT116, left; DLD1, right) and treatment condition (indicated on the right side of each panel)

A bubble plot of the 10 most significantly up- and downregulated GO-terms for biological processes by the treatment groups in both cell lines is presented in Fig. 6. The impact of CBD on biological processes differed between the HCT116 and DLD1 cell lines depending on their respective roles in the cell. Whereas CBD strongly upregulated processes involved in tissue development, filament organization, chemotaxis, and cell migration in the HCT116 cell line, the top upregulated and enriched GO terms in DLD1 were various responses to processes against external stimuli as well as immune system and metabolic processes. Neither CBDA nor the combination CBD/CBDA (1:20) had a significant impact on any biological processes in the HCT116 cells. CBDA treatment resulted in a slight upregulation in DLD1, mainly of metabolic processes addressing biologically active molecules like lipids, hormones, porphyrins, tetrapyrroles, and terpenoids. CBD/CBDA (1:20) showed a moderate upregulation similar to CBDA, primarily of metabolic processes and response to xenobiotic stimulus but also had a minor influence on some additional processes including cell death related processes, such as anoikis. Treatment of HCT116 with the C.s. extract affected, to a certain extent, the same biological processes as CBD, like filament organization, tissue development, and chemotaxis. In contrast, DLD1 cells showed a modest upregulation of metabolic processes affecting retinoids, xenobiotics, terpenoids, and hormones. Downregulated processes were only detected after CBD treatment in HCT116 and DLD1 cells. The most significant downregulated GO terms were shared between both cell lines and include nucleosome assembly, megakaryocyte development, and processes related to protein localization to chromatin or chromosome.

### Hippo signaling pathway is significantly upregulated in HCT116 by CBD

To reveal more information about the impact of CBD, CBDA, CBD/CBDA (1:20), and C.s. extract on cell signaling events, KEGG pathway analysis was performed (Table 1). The DLD1 cell line showed downregulation of signaling pathways affecting alcoholism, neutrophil extracellular trap formation, systemic lupus erythematosus, olfactory transduction, and ribosomes when treated with 10 µM CBD. CBDA treatment did not significantly regulate pathways in DLD1 cells. CBD/CBDA (1:20) treatment resulted in a downregulation of pathways involved in signaling for systemic lupus erythematosus, ribosomes, alcoholism, neutrophil extracellular trap formation, and relaxin signaling. When treated with C.s. extract, DLD1 cells showed a significant downregulation of systemic lupus erythematosus and neutrophil extracellular trap formation pathways. After excluding genes with high FDRs from the pathway analysis, no significantly regulated pathways in DLD1 cells under all treatment conditions compared to the control group were left.

**Table 1:** KEGG pathway analysis of HCT116 and DLD1 cells treated with cannabinoids and C.s. extract

		Direction	KEGG pathways	NES	Genes	Adj.Pval
HCT116	CBD	↓	Systemic lupus erythematosus	−0.7395	84	6.9e-06
			Neutrophil extracellular trap formation	−0.6223	147	9.4e-04
			Alcoholism	−0.6111	153	1.7e-03
		↑	Hippo signaling pathway	0.4933	138	1.8e-03
			Adherens junction	0.557	64	4.4e-02
DLD1	CBD	↓	Alcoholism	−0.6357	149	4.4e-05
			Neutrophil extracellular trap formation	−0.6194	152	1.0e-04
			Systemic lupus erythematosus	−0.6543	85	1.6e-03
			Olfactory transduction	−0.5905	141	2.4e-03
			Ribosome	−0.5615	124	3.6e-02
	CBD/CBDA (1:20)	↓	Systemic lupus erythematosus	−0.7128	85	7.8e-03
			Ribosome	−0.6754	124	7.8e-03
			Alcoholism	−0.6634	149	7.8e-03
			Neutrophil extracellular trap formation	−0.6319	152	2.2e-02
			Relaxin signaling pathway	−0.6536	110	4.6e-02
	C.s. extract	↓	Systemic lupus erythematosus	−0.6948	85	8.6e-03
			Neutrophil extracellular trap formation	−0.6151	152	8.6e-03

Summary of all significantly regulated KEGG signaling pathways identified by gene set enrichment analysis in HCT116 and DLD1 cells treated with 10 μM of either CBD, CBDA, CBD/CBDA (1:20), or C.s. extract compared to control treatment. Significant pathway regulation was calculated using an FDR < 0.05 and log₂ fold change > |1.0|. C.s. ex. = C.s. extract; NES = normalized enrichment score; adj.Pval = adjusted *p*-value corrected for multiple testing

**Fig. 7:**
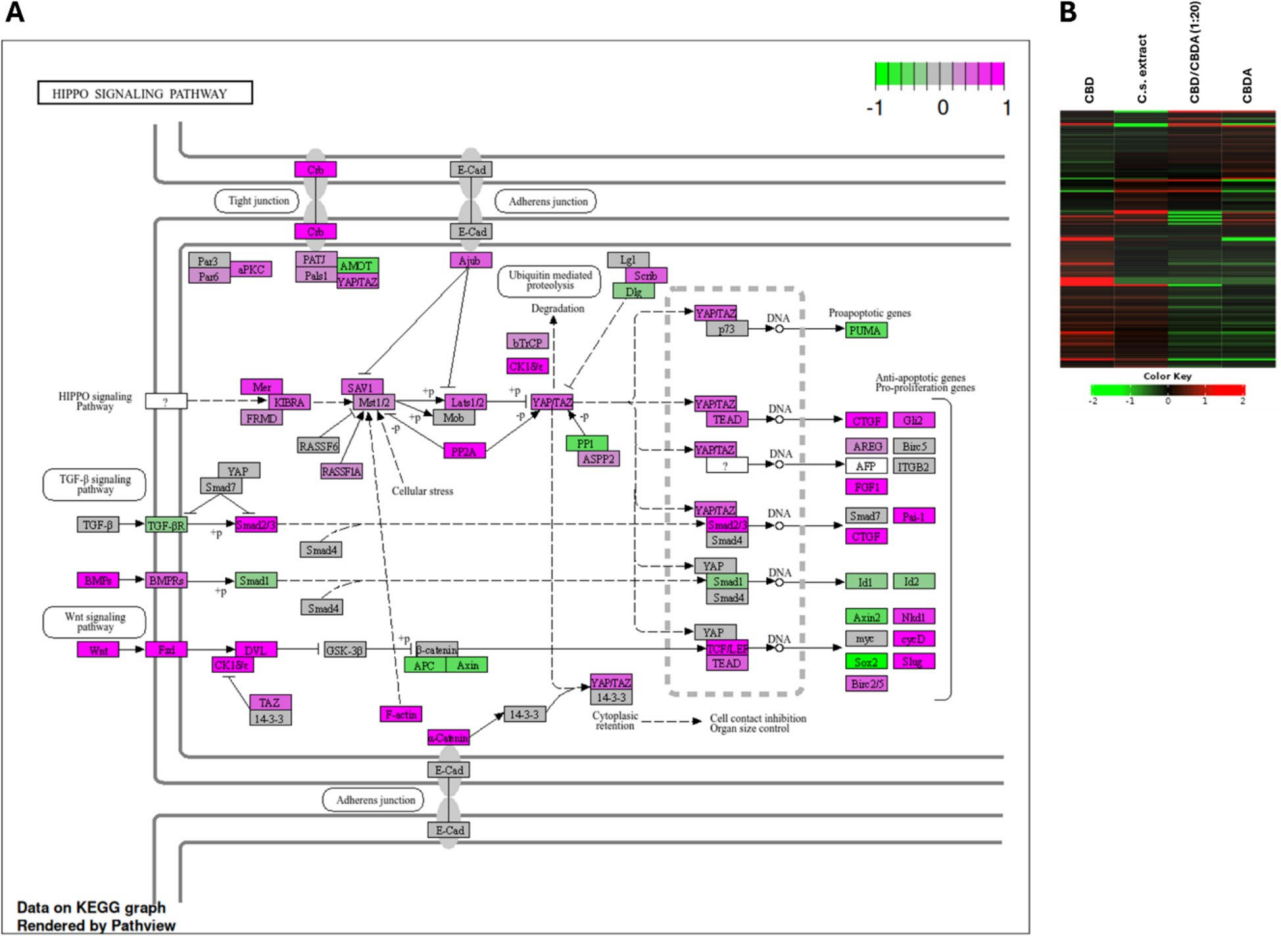
Hippo Signaling pathway in HCT116 cells treated with 10 μM CBD. **A** Gene expression changes within the Hippo signaling pathway in HCT116 cells following treatment with 10 μM CBD compared to control. Magenta-colored genes indicate upregulation, while green-colored genes indicate downregulation. **B** Heatmap representation of gene expression within the Hippo signaling pathway in HCT116 cells treated with 10 μM of either CBD, CBDA, CBD/CBDA (1:20), or C.s. extract. Red bars indicate upregulated genes, and green bars indicate downregulated genes. Note: The color scale of this heatmap is generated directly by the iDEP platform and cannot be modified. Therefore, color-blind–friendly adjustments are not technically possible for this figure

There was a significant downregulation of signaling pathways related to systemic lupus erythematosus, neutrophil extracellular trap formation, and alcoholism in HCT116 cells treated with CBD, along with a significant upregulation of the Hippo signaling pathway and adherens junction pathway. After exclusion of genes with an FDR > 0.05 from pathway analysis, the Hippo signaling pathway remained significantly upregulated in HCT116 cells when treated with 10 µM CBD compared to the control, as illustrated in Fig. 7A.

In the other treatment groups (CBDA, CBD/CBDA (1:20), and C.s. extract), no significant pathway regulation could be detected in the HCT116 cell line.

Figure 7B demonstrates a heatmap of the Hippo signaling pathway of all HCT116 treatment groups, showing a clear upregulation pattern for CBD treatment. While CBDA and CBD/CBDA (1:20) displayed an almost opposing pattern to CBD, the C.s. extract group appeared to slightly upregulate the Hippo signaling pathway. Although treatment with the C.s. extract did not result in significant stimulation, an illustration of the Hippo signaling pathway for C.s. extract shows also several involved genes clearly upregulated. As suggested by the heatmap, Hippo signaling was not affected by treatment with CBDA and CBD/CBDA (1:20) (*Additional File 5*).

## Discussion

In this study, we evaluated the effectiveness of CBDA in two distinct colorectal cancer cell lines and compared its effects with those of CBD. Using a C.s. extract with a 20:1 ratio of CBDA to CBD, we investigated the potential entourage effect of a CBDA-rich C.s. extract by comparing it to a pure CBD/CBDA mixture with the same ratio.

Our results show that CBD had a clearly stronger impact on cell viability and mRNA expression than CBDA in both tested cell lines. In line with this, the IC50 values demonstrated that pure CBD exhibited the highest cytotoxic potency in both cell lines (HCT116: CBD 12.74 < CBD/CBDA (1:20) 15.94 < C.s. extract 20.92 < CBDA; DLD1: CBD 11.32 < CBD/CBDA (1:20) 14.5 < CBDA 18.1 < C.s. extract 23.06), which is consistent with previously reported findings in other cancer cell models (Chen et al., 2024; Lyu et al., 2024; Nahler 2022). Notably, in HCT116 cells, we observed effects of the C.s. extract that were similar to, though weaker than, those of CBD in terms of cell viability, clonogenic growth, and pathway activation. Pathway analysis further revealed differential regulation of biological processes between the two cell lines, suggesting that the impact depends on the cellular genotype. Additionally, the Hippo signaling pathway was significantly upregulated—but only in HCT116 cells—upon treatment with CBD.

Our results from cell viability and clonogenic growth assays are consistent with previous studies investigating the effects of CBDA on cell viability and proliferation, demonstrating lower activity of CBDA compared to CBD, as previously observed in leukemia, breast, and prostate cancer cells (Scott et al., 2013; Petrocellis et al., 2013; Ligresti et al., 2006). Specifically, we observed a significant reduction of cell viability and colony size following CBD treatment in both cell lines compared to the control group. Dose–response assays further revealed that CBD exhibited significantly higher toxicity than CBDA in both cell lines. Interestingly, colony formation assays—commonly considered a stringent measure of cell viability—showed a significant inhibition of colony size by C.s. extract and CBDA, but only in HCT116 cells. However, in DLD1 cells, treatment with 10 µM of CBDA, the CBD/CBDA (1:20) mixture, or the C.s. extract resulted in a significant increase in colony size. These findings suggest that DLD1 cells are not only less sensitive to these compounds but may even exhibit stimulated clonogenic growth when treatment duration does not exceed 24 h. A possible explanation for this growth stimulation is a hormesis-like effect. Hormesis is characterized by a biphasic dose–response, where low doses of a potentially harmful substance or stressor stimulate a beneficial effect, while high doses inhibit function or cause toxicity (Calabrese 2018a; Calabrese and Mattson 2017; Bondy 2023). This phenomenon has been previously described in the context of cannabinoids (Mohammadpour-Asl et al., 2024; Hodges et al., 2020; Calabrese and Rubio-Casillas 2018b).

Due to their distinct genetic backgrounds, the two colorectal cancer cell lines used in this study differ in several molecular features that may influence their responsiveness to phytocannabinoids. HCT116 carries wild-type TP53, displays KRAS G13D activation, and is MLH1-deficient, resulting in a microsatellite-instable (MSI-H) and hypermutated phenotype. In contrast, DLD-1 also exhibits an MSI-H background but harbors a TP53 missense mutation (S241F), KRAS G13D, and activating PIK3CA mutations (Ahmed et al., 2013). These alterations affect stress-response pathways, apoptosis regulation, and downstream RAS/MAPK and PI3K/AKT signaling, all of which have been associated with differential responses to cytotoxic compounds and targeted agents (Yan et al., 2025; Vilar and Tabernero 2013; Voutsadakis 2023; Muradi Muhar et al., 2025), and may therefore contribute to the cell-line-specific effects observed upon treatment with CBDA, CBD, and the C. s. extract.

Differences in cannabinoid sensitivity between cell lines may be further explained by the differential expression of cannabimimetic receptors (Baram et al., 2019). These receptors are part of the endocannabinoid system, which is almost ubiquitously distributed throughout the human body and involved in numerous pathophysiological processes, including neurodegenerative disorders, cardiovascular diseases, and cancer (Lowe et al., 2021). Notably, cannabinoid receptors (CBRs), such as CB1 and CB2, are widely expressed in both normal and cancerous cells. The expression and activation of CBRs, as well as their ability to form heteromers with other receptors, can result in either protective or pathogenic effects, depending on the tumor subtype (Moreno et al., 2020).

Unlike THC, CBD exhibits a very low affinity for CB1 and CB2. However, it interacts with TRP cation channels (TRPV1, TRPV2, TRPM8, and TRPA1), GPR55, 5-HT1A, and PPARγ (Seltzer et al., 2020; Etemad et al., 2022). We found higher mRNA expression levels of CB2, TRPV1, TRPV2, and TRPM8 in HCT116 cells compared to DLD1 cells. These receptor-level variations provide a mechanistic explanation for the stronger responsiveness of HCT116 in viability assays, pathway activation and the observed tendencies toward enhanced extract-mediated effects. DLD1 cells, on the other hand, exhibited a higher PPARγ expression than HCT116 which may underlie the metabolic and proliferative response seen in clonogenic assays, including the hormesis-like stimulation at low doses. Whether a correlation exists between the expression of these cannabinoid-related receptors and CBDA sensitivity in DLD1 and HCT116 cells requires further investigation.

In addition to analyzing different effects of CBD and CBDA on cell viability, our work also aimed to investigate differences between treatment with a pure CBD/CBDA (1:20) mixture or a full-spectrum C.s. plant extract with the same CBD/CBDA ratio. While many in vitro studies report that THC-containing cannabis extracts exert stronger effects than isolated THC, the evidence for analogous effects with CBD is limited (Baram et al., 2019; Comelli et al., 2008; Maayah et al., 2020; Procaccia et al., 2022; Blasco-Benito et al., 2018). Therefore, conclusions regarding enhanced efficacy of CBD-rich extracts should be drawn with caution. The presence of diverse terpenes and cannabinoids in full-spectrum cannabis extract can modulate the binding of endocannabinoids and exogenous cannabinoids, such as THC and CBD, to receptors of the endocannabinoid system (Maayah et al., 2020). This synergistic interaction among multiple compounds of the C.s. plant is referred to as the entourage effect in cannabis research (Christensen et al., 2023). However, the existence and significance of the entourage effect in C.s. plant extracts remain highly debated (Christensen et al., 2023). Previous studies suggest that phytocannabinoid activity can be modulated by co-occurring terpenoids, but the available evidence indicates that such interactions are highly cell-line and chemovar specific. Namdar et al. (2019) showed that in HCT116 cells, certain naturally co-produced terpenoids can enhance the cytotoxicity of CBD (Namdar et al., 2019). In contrast, Romano et al. (2014) found that neither pure CBD nor a CBD-rich botanical extract containing terpenoids affected the viability of HCT116 or DLD1 cells at low micromolar concentrations (Romano et al., 2014). These data demonstrate that potential synergistic effects between cannabinoids and terpenoids cannot be generalized and depend strongly on extract composition, concentration, and cellular context. The C.s. extract used in our study, obtained through low-temperature extraction, contained only trace amounts of CBD while being rich in CBDA but also in terpenes like alpha- and beta-pinen, beta-myrcen, limonen, alpha-cedren, trans-caryophyllen, cis- and trans-nerolidol, and alpha-bisabolol. It is worth noting that the C.s. extract contained cannabichromenic acid (CBCA) at levels comparable to CBD. However, because evidence regarding the biological activity of CBCA is still very limited and its proportion in the extract was relatively low, we consider it unlikely that CBCA substantially contributed to the observed effects. The C.s. extract demonstrated greater cytotoxicity than the CBD/CBDA (1:20) mixture, at least in HCT116 cells. Based on our findings, we propose that the cytotoxic effect of CBDA may be enhanced in the presence of CBD. However, these findings should be interpreted with caution. Since the extract contained a higher total amount of cannabinoids than the CBD/CBDA (1:20) mixture the enhanced efficacy of the extract may simply reflect increased overall cannabinoid bioactivity rather than true synergistic interactions among its constituents. Future studies controlling for total cannabinoid content and testing individual constituents in defined combinations will be required to determine whether CBDA-rich C.s. extracts truly exert entourage or synergistic effects.

Besides assessing clonogenic growth, we analyzed cell adhesion to plastic surfaces in the presence of CBD, CBDA, and C.s. extract using the colony formation assay. Reduced cell adhesion and increased cell migration are characteristics of an invasive tumor phenotype, ultimately leading to metastasis—one of the most critical hallmarks of cancer (Hanahan and Weinberg 2000; Janiszewska et al., 2020). CBD and CBDA have both been associated with inhibiting the migration of breast cancer cells (Takeda et al., 2012; Suttithumsatid et al., 2023). In our study, CBD significantly reduced surface attachment exclusively in DLD1 cells, while no changes were observed in HCT116 cells. Interestingly, CBD/CBDA (1:20) appeared to enhance cell adhesion compared to the control group. Further studies investigating cell adhesion are required to fully understand the impact of CBDA on colorectal cancer cells.

RNA sequencing enabled a more detailed characterization of the molecular effects exerted by CBD and CBDA on CRC cells. Overall, the number of DEGs in both cell lines following treatment with 10 µM of either CBD, CBDA, CBD/CBDA (1:20), or C.s. extract for 48 h was relatively low. In brief, our data indicate that CBD exerted the strongest impact on gene expression, whereas CBDA had the least effect. Comparing the C.s. extract with CBD/CBDA (1:20) revealed an increase in DEGs with the extract, at least in HCT116, suggesting a possible entourage effect.

Focusing on the DEGs, ANGPTL4 and CYP1A1 were found to be upregulated in almost all treatment groups of both cell lines. ANGPTL4 has been shown to promote the proliferation and migration of CRC cells, and its protein expression correlates positively with CRC stage, indicating a potential role in metastasis (Zhang et al., 2022). CYP1A1, on the other hand, is a member of the Cytochrome P450 superfamily of enzymes, which are involved in the primary metabolism of cannabinoids (Hryhorowicz et al., 2018). Another important enzyme family involved in drug metabolism and detoxification is the UDP-glucuronosyltransferase (UGT) family. UGTs have also been identified as key catalysts in cannabinoid metabolism (Hryhorowicz et al., 2018). In our RNA sequencing study, we found that UGT1A1 was significantly upregulated in all treatment groups of DLD1 cells. Additionally, DHRS3, which is ubiquitously expressed across various tissues and catalyzes the oxidation and reduction of a broad range of substrates, including retinoids (Lundová et al., 2015), was also upregulated in all four treatment groups of DLD1 cells.

In contrast, HCT116 cells exhibited a significant downregulation of the ARRDC4 gene in all tested treatment groups. ARRDC4 has been reported to be downregulated in CRC tissue and has been implicated in the Hippo pathway by promoting the degradation of its key effector, Yes-associated protein-1 (YAP1) (Huang et al., 2023). Together with Tafazzin (TAZ), YAP1 interconnects the Hippo signaling pathway with other key signaling cascades, including those mediated by Wnt growth factors and transforming growth factor β (TGF-β). These pathways regulate essential biological processes such as organ development, epithelial homeostasis, tissue regeneration, wound healing, and immune modulation by controlling cell growth, proliferation, and apoptosis (Dey et al., 2020).

The activity and subcellular localization of the YAP1/TAZ complex are regulated by upstream signals from the extracellular matrix, mechanical forces, cell adhesion, cell polarity, mitogens, tyrosine kinase receptors, G protein-coupled receptors, and alterations in cellular metabolism (Dey et al., 2020). Although we did not observe any significant changes in YAP1 or TAZ expression in our samples (data not shown), KEGG pathway analysis revealed a significant upregulation of the Hippo signaling pathway in HCT116 cells following exposure to CBD. Additionally, we observed an upregulation of the Wnt pathway, with significant expression of Wnt-related RNAs, particularly Wnt9a, Wnt7a, and Wnt7b, leading to the upregulation of Wnt target genes.

Conversely, treatment with CBDA, CBD/CBDA (1:20), and C.s. extract in HCT116, as well as all four treatment groups in DLD1, did not result in significant changes in KEGG pathway analysis after refinement. However, we observed an upregulation of effector genes of the Wnt and Hippo signaling pathways in HCT116 cells treated with C.s. extract, similar to the CBD-treated group, albeit without reaching statistical significance.

The upregulation of Wnt and Hippo signaling by CBD is inconsistent with the growth inhibition we observed in the viability assays. Nallathambi et.al. also reported cytotoxic activity and a reduction in differentially expressed genes related to Wnt signaling in CRC cells after treatment with THCA- and CBGA- rich fractions of C.s. extract (Nallathambi et al., 2018). Additionally, other studies support our findings regarding the relationship between cannabinoids and Wnt signaling. In neurodegenerative diseases, CBD has been shown to increase Wnt/β-catenin signaling by decreasing glycogen synthase kinase-3 β (GSK-3β) activity, a key negative regulator of the Wnt/β-catenin pathway, thereby reducing inflammatory processes (Renard et al., 2017; Vallée et al., 2021a; Vallée et al., 2017; Vallée et al., 2021b). Further studies analyzing the Wnt and Hippo signaling pathways and their target genes at both the mRNA and protein levels are necessary to clearly determine the impact of CBD or C.s. extracts on CRC cells.

The top 10 GO terms for biological processes regulated in HCT116 cells by CBD treatment included mechanisms primarily involved in cell differentiation, polarity, adhesion, and motility which are partially controlled by Wnt and Hippo signaling (Dey et al., 2020; Loh et al., 2016). Some of those biological processes were also upregulated in HCT116 cells following exposure to the C.s. extract. In contrast, treatment with CBDA or CBD/CBDA (1:20) had no significant impact on biological processes in HCT116 cells, indicating a strong effect of CBD which is contained in a small proportion in the C.s. extract and supporting a potential entourage effect of the C.s. extract.

DLD1 cells responded differently to treatment with the C.s. extract and cannabinoids compared to HCT116. CBD treatment primarily induced biological processes related to the cellular response to external stimuli, whereas CBDA, CBD/CBDA (1:20), and C.s. extract activated a broad range of metabolic processes, including those related to lipids, hormones, and flavonoids. The induction of these metabolic processes may reflect the biochemical degradation and metabolism of CBD and CBDA within DLD1 cells. Nevertheless, we identified some similarities between both cell lines: When exposed to CBD, biological processes related to chromatin and chromosome organization were downregulated, consequently leading to decreased cell division, which is consistent with our viability results.

## Conclusion

In conclusion, our data demonstrate that CBD exerts stronger effects on CRC cell viability and gene expression than CBDA in the tested cell lines. In contrast, CBDA showed only limited activity, suggesting that CBD represents a more promising compound for further investigation in CRC.

The CBDA-rich C.s. extract exhibited greater efficacy than the corresponding CBD/CBDA (1:20) mixture in HCT116 cells. However, this effect cannot be unequivocally attributed to a true entourage effect and may instead reflect additive effects or increased overall cannabinoid content. Future studies controlling for total cannabinoid levels and incorporating additional time points will be required to distinguish additive from synergistic interactions and to better understand the dynamic cellular responses to cannabinoid treatment.

## Supplementary Information

Additional file 1.

Additional file 2.

Additional file 3.

Additional file 4.

Additional file 5.

## Data Availability

The datasets used and/or analyzed during the current study are available from the corresponding author on reasonable request. RNA-seq reads are publicly available at the NCBI Sequence Read Archive under the BioProject accession PRJNA1178466.

## Abbreviations

CBD: Cannabidiol
CBDA: Cannabidiolic acid
CBCA: Cannabichromenic acid
C.s.: Cannabis sativa
CRC: Colorectal cancer
THC: Δ-9- Tetrahydrocannabinol
COX: Cyclooxygenase
HPLC: High-performance liquid chromatography
GC: Gas chromatography
FID: Flame ionization detection
FCS: Fetal calf serum
PBS: Phosphate-buffered saline
RIN: RNA integrity numbers
RNA-seq: RNA sequencing
GO: Gene ontology
FDR: False discovery rate
Log_2_FC: Log_2_ fold change
KEGG: Kyoto encyclopedia of genes and genomes
iDEP: Integrated differential expression and pathway analysis
SD: Standard deviation
IC50: Half maximal inhibitory concentration
PC: Principal component
PCA: Principal component analysis
DEG: Differentially expressed genes
TPM: Transcripts per million
CPA4: Carboxypeptidase 4
ARRDC4: Arrestin domain-containing 4
KRT7: Keratin 7
CYP1A1: Cytochrome P450 family 1 subfamily A member 1
ANGPTL4: Angiopoietin-like 4
UGT1A1: UDP glucuronosyltransferase family 1 member A1
PDK4: Pyruvate dehydrogenase lipoamide kinase isozyme 4
NRP2: Neuropilin 2
DHRS3: Dehydrogenase/reductase 3
AOAH: Acyloxyacyl hydrolase
RIMBP3B: RIMS binding protein 3B
DAPK2: Death-associated protein kinase 2
AHRR: Aryl hydrocarbon receptor repressor
CBR: Cannabinoid receptors
TRP: Transient receptor potential
GPR55: G protein-coupled receptor 55
5-HT1A: Serotonin 1A receptor
PPARγ: Peroxisome proliferator-activated receptor gamma
NES: Normalized enrichment score
adj.Pval: Adjusted p-value
UGT: UDP-glucuronosyltransferase
DHRS3: Dehydrogenase/Reductase 3
YAP1: Yes-associated protein-1
TAZ: Tafazzin
TGF-β: Transforming growth factor β
GSK-3β: Glycogen synthase kinase-3 β
MSI-H: Microsatellite-instable
