# Supplementary material for: The differential effects of CBD and CBDA on viability and mRNA expression in colorectal cancer cells
Source: J Cannabis Res. 2026 Jan 16;8:24. doi: 10.1186/s42238-026-00391-2 (PMC12895959; doi:10.1186/s42238-026-00391-2)
Supplement: Supplementary file 1 — Additional file 1. [file 42238_2026_391_MOESM1_ESM.pdf]

Additional File 1a Cannabinoids of C.s. extract

Cannabinoid	Result [% solids]
**CBD** cannabidiol	**0.137**
**CBDA** cannabidiolic acid	**2.45**
**T-CBD** total cannabidiol (CBD+CBDA)	**2.29**
**THCΔ8** Δ-8-tetrahydrocannabinol	**<0.010**
**THCΔ9** Δ-9-tetrahydrocannabinol	**0.012**
**THCA** tetrahydrocannabinolic acid	**0.099**
**T-THC** total tetrahydrocannabinol (THC+THCA)	**0.099**
**CBG** cannabigerol	**0.014**
**CBGA** cannabigerolic acid	**0.079**
**T-CBG** total cannabigerol (CBG+CBGA)	**0.083**
**CBDV** cannabidivarin	**<0.010**
**CBDVA** cannabidivarinic acid	**<0.010**
**THCV** tetrahydrocannabivarin	**<0.010**
**THCVA** tetrahydrocannabivarinic acid	**<0.010**
**CBN** cannabinol	**<0.010**
**CBNA** cannabinolic acid	**<0.010**
**CBL** cannabicyclol	**<0.010**
**CBC** cannabichromene	**<0.010**
**CBCA** cannabichromenic acid	**0.13**

All cannabinoids identified in the C.s. extract were analyzed by HPLC with UV/VIS detection. Results are expressed as in % of total solids.

Additional File 1b Terpenoids of C.s. extract

Terpenoids	Result [mg/kg solids]
alpha-Pinen	150
Camphen	< 10
Sabinen	< 10
beta-Pinen	27
beta-Myrcen	190
alpha-Phellandren	< 10
(+)-d-3-Caren	< 10
alpha-Terpinen	< 10
Limonen	21
trans-beta-Ocimen	< 10
Eucalyptol	< 10
cis-beta-Ocimen	< 10
gamma-Terpinen	< 10
Terpinolen	< 10
Sabinenhydrat	< 10
Linalool	< 10
(+,-)-Fenchon	< 10
(+)-endo-Fenchylalkohol	11
Isopulegol	< 10
(+,-)-Camphor	< 10
Isoborneol	< 10
Menthol	< 10
(+,-)-Borneol	< 10
alpha-Terpineol	< 10
gamma-Terpineol	< 10
Nerol	< 10
Pulegon	< 10
Geraniol	< 10
Geranylacetat	< 10
Farnesen	< 10
alpha-Cedren	85
trans-Caryophyllen	51
alpha-Humulen	< 10
Valencen	< 10
cis-Nerolidol	67
trans-Nerolidol	16
(-)-Guaiol	< 10
Caryophyllenoxid	< 10
Cedrol	< 10
(-)-alpha-Bisabolol	90

All terpenoids identified in the C.s. extract were analyzed by GC with FID detection. Results are expressed as in mg/kg of total solids.
