# Supplementary material for: The differential effects of CBD and CBDA on viability and mRNA expression in colorectal cancer cells
Source: J Cannabis Res. 2026 Jan 16;8:24. doi: 10.1186/s42238-026-00391-2 (PMC12895959; doi:10.1186/s42238-026-00391-2)
Supplement: Supplementary file 5 — Additional file 5. [file 42238_2026_391_MOESM5_ESM.pdf]

CBDA

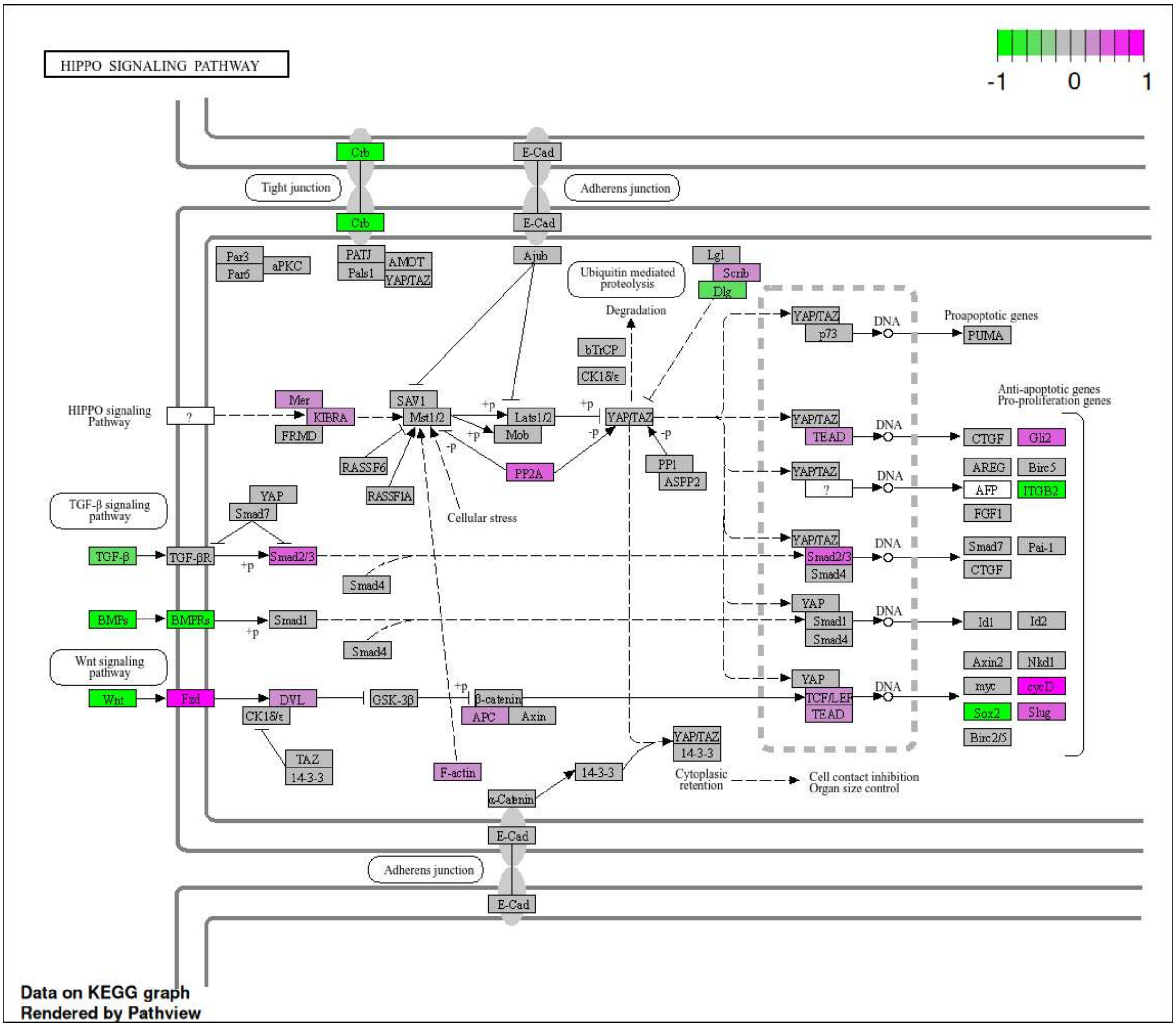

Data on KEGG graph
Rendered by Pathview

CBD/CBDA (1:20)

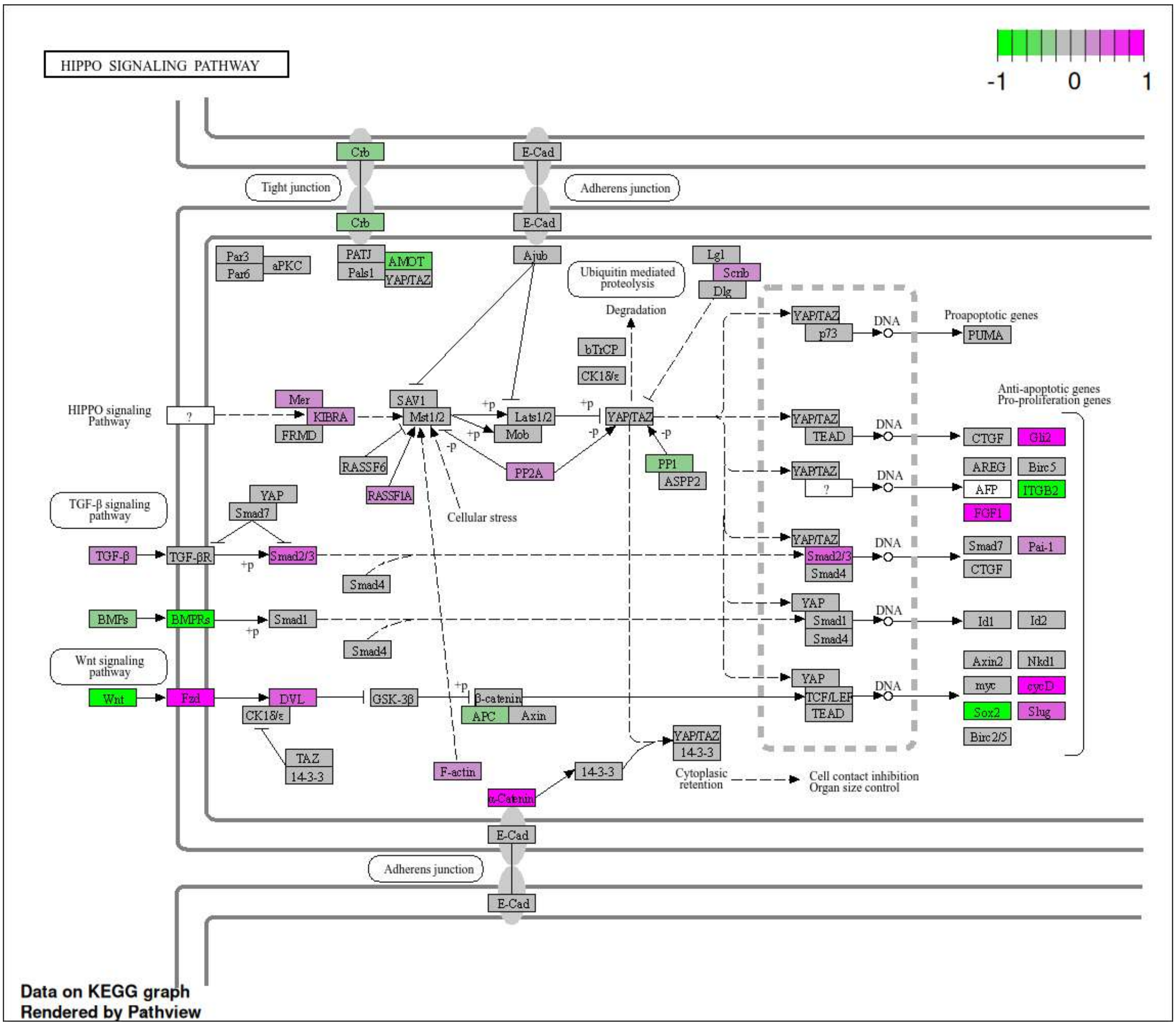

HIPPO SIGNALING PATHWAY
-1
0
1
Crb
E-Cad
Tight junction
Adherens junction
Crb
E-Cad
Par3
Par6
aPKC
PATJ
Pals1
AMOT
YAP/TAZ
Ajub
Lgl
Scrib
Dlg
Ubiquitin mediated proteolysis
Degradation
YAP/TAZ
p73
DNA
Proapoptotic genes
PUMA
bTrCP
CK18/ε
Anti-apoptotic genes
Pro-proliferation genes
HIPPO signaling Pathway
?
Mer
KIBRA
FRMD
SAV1
Mst1/2
Lats1/2
Mob
YAP/TAZ
YAP/TAZ
TEAD
CTGF
Gli2
RASSF6
RASSF1A
PP2A
PP1
ASPP2
Cellular stress
AREG
Birc5
AFP
ITGB2
FGF1
TGF-β signaling pathway
YAP
Smad7
TGF-β
TGF-βR
Smad2/3
Smad4
Smad7
Pai-1
CTGF
BMPs
BMPRs
Smad1
Smad4
YAP
Smad1
Smad4
Id1
Id2
Wnt signaling pathway
Wnt
Fzd
DVL
CK18/ε
GSK-3β
β-catenin
APC
Axin
TCF/LEF
TEAD
Axin2
Nkd1
myc
cycD
Sox2
Slug
Birc2/5
TAZ
14-3-3
F-actin
α-Catenin
14-3-3
YAP/TAZ
14-3-3
Cytoplasic retention
Cell contact inhibition
Organ size control
E-Cad
Adherens junction
E-Cad
Data on KEGG graph
Rendered by Pathview

C.s. extract

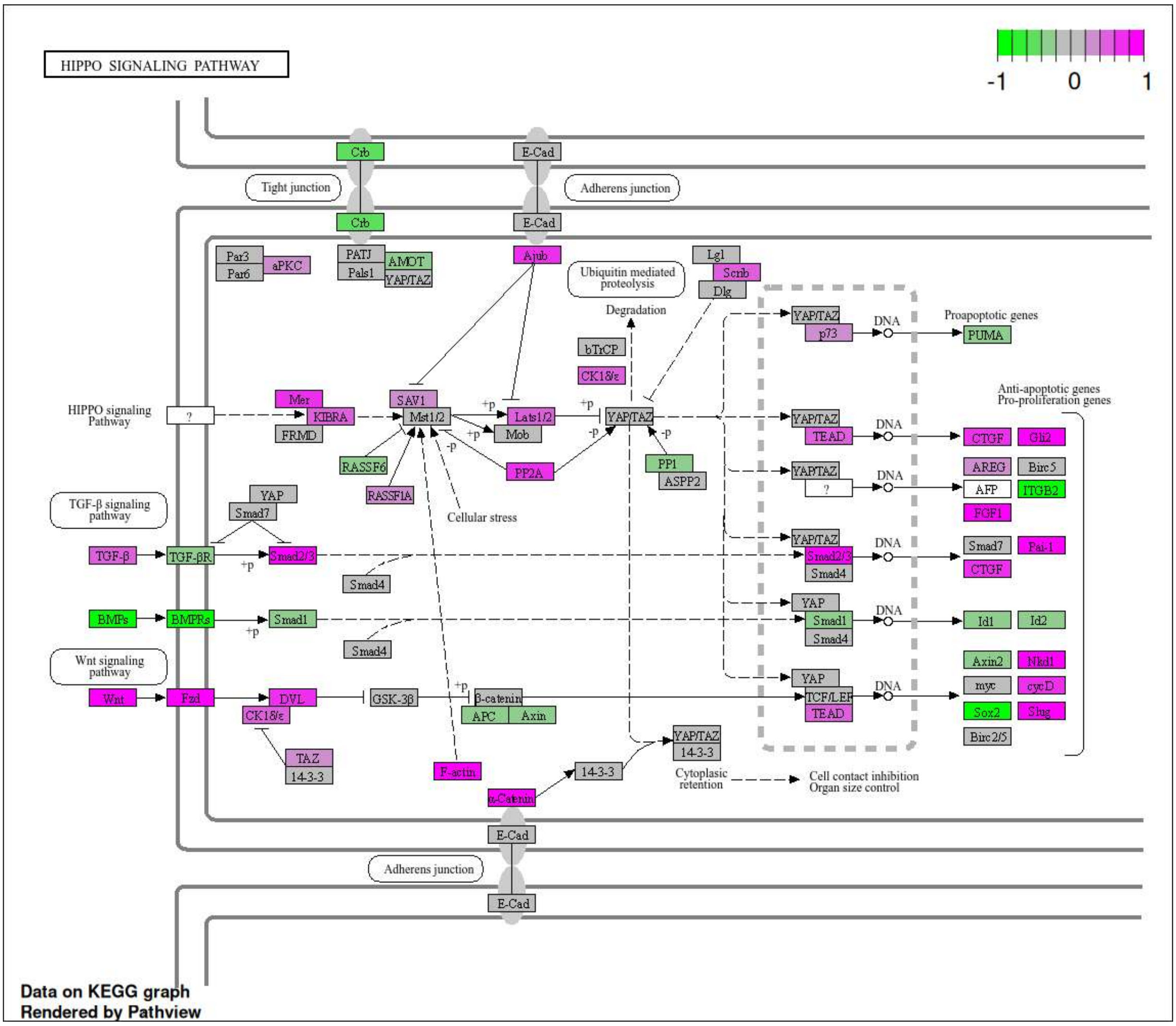

Additional File 5 Hippo Signaling pathway in HCT116 cells treated with CBDA, CBD/CBDA (1:20), or C.s. extract. Gene expression changes within the Hippo signaling pathway in HCT116 cells following treatment with 10 μM CBDA, CBD/CBDA (1:20), or C.s. extract compared to control. Magenta indicates upregulated genes, while green indicates downregulated genes.
